# Recovery Trajectories of Motor Function After Hip Fracture Surgery in Older Patients: A Multicenter Growth Mixture Modeling Study in Acute Care Hospitals

**DOI:** 10.3390/geriatrics10060167

**Published:** 2025-12-15

**Authors:** Keisuke Nakamura, Yasushi Kurobe, Keita Sue, Shuhei Yamamoto, Kimito Momose

**Affiliations:** 1 Department of Physical Therapy, School of Health Sciences, Shinshu University, Matsumoto 390-0802, Japan; kmomose@shinshu-u.ac.jp; 2 Department of Rehabilitation, Fujimi-Kogen Hospital, Fujimi-Kogen Medical Center, Fujimi, Suwa District, Nagano 399-0214, Japan; yasushi1818@gmail.com; 3 Department of Rehabilitation, JA Nagano Kouseiren, Kakeyu-Misayama Rehabilitation Center Kakeyu Hospital, Ueda, Nagano 386-0396, Japan; keitasue3314@gmail.com; 4 Department of Rehabilitation, Shinshu University Hospital, Matsumoto 390-8621, Japan; syamamoto@shinshu-u.ac.jp

**Keywords:** hip fracture, functional recovery trajectory, growth mixture modeling

## Abstract

**Background/Objective**: Hip fractures in older adults are a major public health concern due to their high rates of morbidity, mortality, and long-term disability. Although surgical and postoperative care have improved, recovery outcomes remain highly variable. Identifying early functional recovery patterns could support individualized rehabilitation and discharge planning. This study aimed to identify distinct early recovery trajectories of motor function within 30 days after hip fracture surgery using growth mixture modeling (GMM) and to examine patient- and hospital-level factors associated with these patterns. **Methods**: A retrospective cohort study was conducted using data from the Nagano Hip Fracture Database, including 2423 patients aged ≥65 years across 17 acute care hospitals in Japan (2019–2024). Functional recovery was measured using the motor subscale of the Functional Independence Measure (FIM-motor) at 0, 7, and 28 days post-admission. Latent trajectory model was used to identify distinct recovery patterns. Multinomial logistic regression analyzed predictors of class membership. **Results**: Three recovery trajectories were identified: high/rapid improvement (26.7%), intermediate (32.6%), and poor/flat recovery (40.7%). Older age, cognitive impairment, and lower baseline mobility were strongly associated with membership in the poor-recovery class. Early trajectory classes significantly predicted discharge outcomes, including FIM-motor scores and discharge destination. Sensitivity analysis confirmed the robustness of findings, with minimal impact from hospital-level clustering. **Conclusions**: Distinct early recovery trajectories exist after hip fracture surgery and are strongly influenced by baseline cognitive and functional status. Early identification of recovery patterns can enhance personalized rehabilitation and inform discharge planning, offering valuable insights for clinical practice.

## 1. Introduction

Hip fracture is a major health problem in aging societies and is associated with high morbidity, mortality, and functional decline [1,2,3]. Despite advances in surgical techniques and postoperative care, many older adults fail to regain their prefracture mobility levels, resulting in prolonged disability, institutionalization, and increased healthcare costs. Functional outcomes after hip fractures are highly heterogeneous and are influenced by factors such as age, comorbidities, cognitive impairment, fracture type, and timing of ambulation initiation [4,5,6,7,8,9,10,11].

The early identification of patients at risk of poor recovery is crucial for optimizing rehabilitation strategies and discharge planning. Traditional prognostic studies have focused on baseline characteristics and single-time point outcomes; however, these approaches may overlook the dynamic and nonlinear nature of functional recovery. Trajectory analysis offers an alternative framework to capture heterogeneous recovery patterns over time. Growth mixture modeling (GMM) is particularly suited for this purpose, as it allows the identification of latent subgroups that follow distinct functional trajectories while accounting for within-patient variability.

Previous studies that applied trajectory models to hip fracture rehabilitation were limited in scope. Tseng et al. identified three distinct trajectories of long-term activities of daily living (ADL) recovery over 2 years in a single-center randomized trial [12], whereas Salpakoski et al. reported two trajectories of walking recovery during a 10-week follow-up in a small cohort and reported entropy as a measure of classification quality [13]. More recently, a multicenter study characterized functional trajectories using the GMM [14]; however, these prior studies predominantly examined long-term outcomes, were often conducted in single-center settings, or focused on mobility measures rather than comprehensive ADL function. As a result, the heterogeneity of short-term postoperative recovery in real-world multicenter acute care settings remains poorly understood.

Evidence is particularly limited regarding whether early (≤30 days) functional trajectories can be empirically identified across multiple hospitals using standardized Functional Independence Measure(FIM)-motor assessments, and how such trajectories may relate to baseline characteristics and early discharge outcomes. Addressing this gap is essential because the acute postoperative phase is the most dynamic period of functional change and directly influences rehabilitation planning and discharge pathways.

In contrast, the present study leveraged a multicenter cohort of 17 hospitals and focused on ADL recovery assessed using the motor FIM within the first 30 days after surgery. This early period represents the most dynamic phase of functional change and is critical for discharge planning and early rehabilitation.

The objective of this study was to identify distinct early recovery trajectories in motor function within 30 days after hip fracture surgery using growth mixture modeling and to examine both patient- and hospital-level factors associated with these patterns. We hypothesized that multiple recovery trajectories exist and that these trajectories are significantly associated with cognitive status, age, and preinjury mobility.

## 2. Materials and Methods

### 2.1. Study Design and Setting

This study was a retrospective analysis of prospectively collected data from the Nagano Hip Fracture Database, a multicenter registry in Japan that gathers information on patient characteristics and rehabilitation outcomes [7]. For this study, we included consecutive patients from 17 acute care hospitals between 1 December 2019 and 31 December 2024. Data were collected using a secure cloud-based electronic data-capture system.

### 2.2. Participants

Consecutive patients with femoral neck or trochanteric fractures who underwent surgery were eligible. The inclusion criteria were as follows: (1) age ≥ 65 years, (2) admission to an acute care hospital, and (3) no postoperative weight-bearing restrictions. Wheelchair users were included if the other criteria were met. Patients who died during hospitalization were excluded.

### 2.3. Bias Minimization

To reduce the selection bias, all eligible patients were consecutively included in the study without sampling. Information bias was minimized through standardized data collection protocols and training across participating hospitals.

### 2.4. Sample Size

No formal sample size calculation was performed, as this was a consecutive all-case registry study involving all eligible admissions across 17 hospitals over a 5-year period. The large sample size (n = 2423) provides sufficient statistical power for trajectory modeling and subgroup analyses. While the sample size requirements for latent variable models depend on factors such as model complexity and effect size, Monte Carlo studies provide clear benchmarks [15]. For growth models, a class of trajectory models, the sample size required to achieve 80% power under the most stringent conditions (small effect size of 0.1 and missing data) was 1025. Since our sample substantially exceeds this benchmark, we are confident of the high statistical power required to detect small effects and identify potentially small unobserved subpopulations.

### 2.5. Ethics

This study was approved by the Ethics Committee of the School of Medicine, Shinshu University, on 12 November 2019 (protocol number: 4541), and conducted in accordance with the principles of the Declaration of Helsinki. Given the observational nature and retrospective analysis of prospectively collected data, the requirement for written informed consent was waived, and an opt-out procedure was performed at each hospital. The study information is publicly available through the University Hospital Information Network (UMIN-CTR, UMIN000054114). Also, this study was conducted according to the Strengthening the Reporting of Observational Studies in Epidemiology (STROBE) guidelines for cohort studies [16].

### 2.6. Measurements

The patient’s background data included age, sex, body mass index (BMI), comorbidities (respiratory, cardiovascular, and neurological diseases), cognitive function before the fracture, pre-injury residence, and mobility status. Cognitive function was assessed using the Degree of Daily Life Independence Score for People with Dementia (DDLIS-PD) [17,18]. This assessment consists of seven scales and is widely used to evaluate dementia in Japan. In this study, cognitive impairment was defined as DDLIS-PD grade II, with independence from some hindrances, or higher grades. We have previously applied this scale in a hip fracture cohort [7], demonstrating its clinical relevance by showing a significant association between higher DDLIS-PD grades and reduced likelihood of early postoperative mobilization. Although formal validation in post-fracture populations remains limited, its practicality and prior clinical use support its application in this study.

Mobility (preinjury and at discharge) was categorized as follows: (1) no aid, (2) cane, (3) walker, (4) other aid, or (5) wheelchair. Residence (before admission and at discharge) was categorized as: (1) home, (2) facility, (3) rehabilitation hospital, (4) long-term care hospital, (5) other, or (6) death.

Additional data included fracture type, surgical procedure, perioperative complications (including deep vein thrombosis, peroneal nerve palsy, infection, and falls), number of days from admission to rehabilitation, and number of days to first ambulation.

Functional recovery was measured using the FIM [19], which includes total, motor (FIM-motor), and cognitive subscores. FIM assessments were conducted on the day after surgery (postoperative day 1); at 1 week, 4 weeks, 8 weeks, and 12 weeks; and at discharge using standardized procedures across all participating hospitals.

### 2.7. Statistical Analysis

All analyses were conducted using R software (version 2.7). To address potential sources of selection bias, all eligible patients across the 17 participating hospitals were included consecutively. Measurement bias was minimized using standardized data collection protocols, trained assessors, and validated tools such as the FIM and DDLIS-PD.

Missing baseline/background covariates were imputed using missForest [20] with 100 trees (ntree = 100) and a fixed random seed (1234) for reproducibility. This is a nonparametric random-forest-based method suitable for mixed-type data. The imputation set included age, sex, BMI, cognitive impairment, fracture type, respiratory/cardiac/neurological comorbidities, residence, and indoor/outdoor walking status. Outcome variables (FIM scores and class membership) were not imputed to avoid distorting the recovery patterns.

To model early recovery patterns, we applied GMM using the lcmm package [21]. Motor FIM (FIMmotor) trajectories were modeled using days since operation as the time scale (1, 7, and 28 days). Motor FIM (FIMmotor) scores were modeled using postoperative days as the time scale (POD1, POD7, POD28). This 30-day window was selected because it represents the most dynamic phase of recovery, during which most patients remain in acute care. Assessments were conducted at standardized time points on POD1, POD7, and POD28.

Model selection was based primarily on the Bayesian Information Criterion (BIC), with additional consideration of entropy and average posterior probabilities (APP) to evaluate classification precision. We tested models with 1 to 4 latent classes using both linear and quadratic specifications and found that the three-class quadratic model provided the best fit (lowest BIC) and acceptable classification quality (entropy > 0.6, APP > 0.8) [22,23,24]. The model specification included random intercepts and slopes for time at the subject level, class-specific fixed effects, and class-specific residual variances. Each patient was assigned to the class with the highest posterior probability.

In the sensitivity analysis, we introduced hospitals (17 facilities) as a clustering factor in the growth mixture model using the cluster argument in the lcmm package, which adjusts standard errors for within-cluster correlation rather than modeling random effects. This approach accounts for potential intra-hospital similarities (e.g., clinical protocols and rehabilitation resources) without altering the estimated fixed effects or class structures. The aim was to examine the robustness of the identified trajectory classes under possible institutional clustering.

In a second sensitivity analysis, we repeated the latent class modeling using only individuals with complete FIM-motor data at postoperative day 28 (n = 989), applying the same model specification and class structure. This aimed to assess potential bias due to early transfer or loss of follow-up before day 28.

To further explore institutional effects, we compared class distributions across hospitals using cross-tabulations and χ^2^ tests, with results visualized as stacked bar plots. Because smaller hospitals contributed fewer cases, we interpreted facility-specific proportions with caution, recognizing that the observed variation may partly reflect a random case mix.

Baseline characteristics and discharge outcomes were compared across classes using the Kruskal–Wallis test for continuous variables and χ^2^ or Fisher’s exact tests for categorical variables, with Holm-adjusted post hoc pairwise tests. The determinants of class membership were further examined using multinomial logistic regression, with Class 2 (intermediate trajectory) as a reference.

## 3. Results

**Figure 1:**
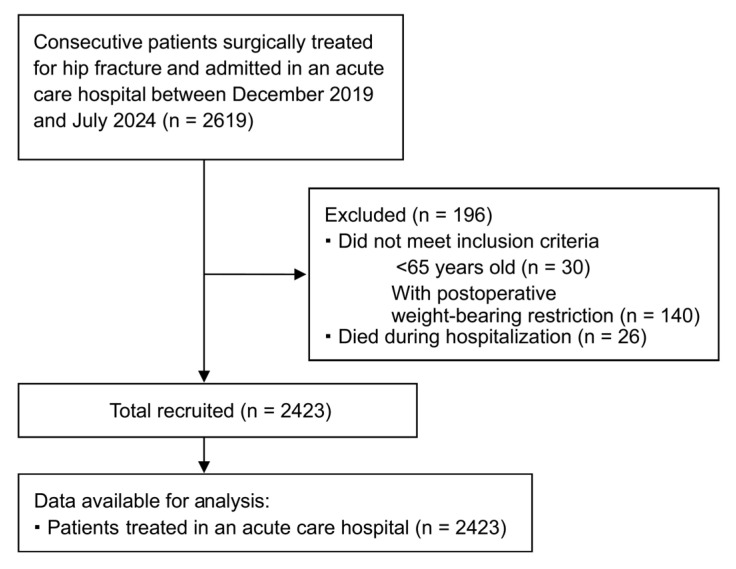
Flow diagram of patient selection for the study cohort.

A patient flow diagram illustrating the inclusion, exclusion, and final analytical samples is shown in Figure 1. We analyzed 2423 admissions. The median age was 87 years (IQR, 81–92 years), and the median length of stay (LOS) was 29 days (range, 20–46 days). The median discharge FIM-motor score was 53 (33–69) and the FIM-total score was 79 (54–100). The distributions of baseline characteristics and FIMs are summarized in Table 1.

### 3.1. Latent Trajectory Model (≤30 Days)

**Figure 2:**
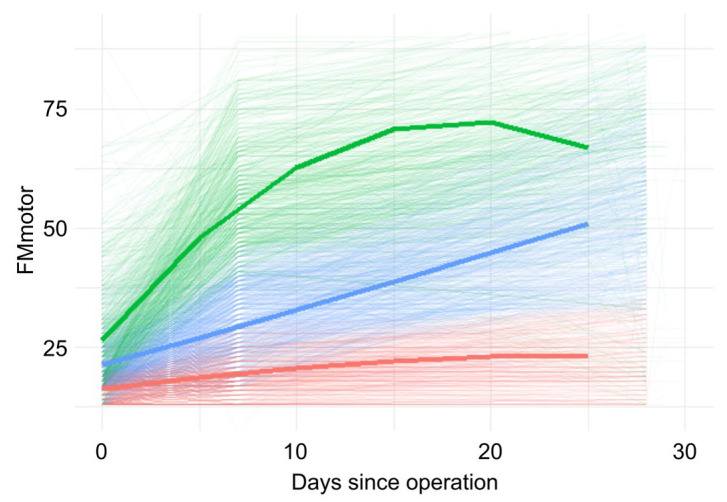
Individual FIM-motor trajectories during the first 30 days after surgery, with estimated class trajectories overlaid.

A three-class quadratic model provided the best fit (BIC = 54,110.1) versus the 1-class, 2-class, and 4-class alternatives (Supplementary Table S1). The class proportions were 26.7%, 32.6%, and 40.7% for Classes 1–3, respectively. Although the log-likelihood improved with an increasing class number, the 3-class quadratic model had the lowest BIC (54,110.1), supporting it as the optimal solution. Model classification quality was also acceptable, with an entropy of 0.607 and class-specific average posterior probabilities (APPs) of 0.901 (Class 1), 0.825 (Class 2), and 0.833 (Class 3), all exceeding the predefined thresholds of entropy > 0.6 and APP > 0.8. These values indicate a satisfactory degree of separation and classification precision between the latent classes. The figure shows distinct patterns: a high/rapid improvement class (class1), an intermediate/linear improvement class (class2), and a low/flat class (class3) (Figure 2). The model fit indices and class proportions are reported in the lcmm summary.

In the sensitivity analysis, which included hospitals as a clustering factor, the model fit indices (loglikelihood and BIC) were similar to those in the primary analysis (Supplementary Table S1). The class proportions were unchanged, apart from the permutation of Class 1 and Class 2 labels, and the estimated recovery trajectories were virtually identical. Entropy slightly improved in the facility-adjusted model (0.623), further supporting model robustness. These results confirm that the findings are robust and not driven by interhospital variations. In a second sensitivity analysis restricted to participants with available FIM-motor scores at postoperative day 28 (n = 989), the same 3-class quadratic model structure yielded similar class proportions (31.5%, 40.7%, and 27.9% for Classes 1–3) and improved entropy (0.676), with APPs of 0.844, 0.835, and 0.883, respectively. These results further reinforce the stability of the class solution and classification accuracy even under more conservative data inclusion criteria.

- Green (Class 1): High initial FIM-motor scores with rapid early improvement, followed by a plateau.
- Blue (Class 2): Moderate initial scores with steady linear improvement throughout the postoperative period.
- Red (Class 3): Low initial scores with minimal functional gain over time.

### 3.2. Hospital-Level Class Distribution

Class distributions differed significantly across hospitals (χ^2^ = 131.7, df = 32, *p* < 0.001). Some institutions had a higher proportion of patients in the high recovery class, while others had more patients in the poor recovery class (Supplementary Figure S1). However, the proportions were influenced by sample size, with smaller hospitals showing more unstable distributions.

### 3.3. Baseline Characteristics by Class

The baseline characteristics differed across classes (Table 2). In brief, patients in Class 1 were younger (median 82 [74, 88] years) and more often cognitively intact, while Class 3 patients were the oldest (median 90 [86, 94] years) and had the highest prevalence of cognitive impairment and intertrochanteric fractures. Functional status at admission also differed across the indoor/outdoor walking categories (all global and pairwise comparisons were significant after Holm correction; Table 2).

### 3.4. Multivariable Correlates of Class Membership

In the multinomial logistic models (reference = Class 2), age was not significantly associated with membership in the lower trajectory group (Class 3) (OR 1.01 per year increase, 95% CI 1.00–1.03, *p* = 0.187). Cognitive impairment showed a strong association with lower trajectory membership (OR 2.80, 95% CI 2.14–3.66). For Class 1 versus Class 2, younger age (OR 0.95 per year, 95% CI 0.94–0.96, *p* = 0.002), better indoor walking ability, and absence of cognitive impairment were associated with membership in the higher functional trajectory group. The full adjusted odds ratios and 95% CIs are shown in Table 3.

### 3.5. Discharge Outcomes by Class

Discharge function and destination differed markedly (Table 4). The median FIM-motor scores at discharge were 68 (58–79) in Class 1, 48.5 (38–62) in Class 2, and 25 (19–36) in Class 3 (all *p* < 0.001). Discharge to home occurred in 32.9% (Class 1), 15.5% (Class 2), and 7.8% (Class 3) of patients; wheelchair use at discharge was 17.1%, 52.5%, and 84.5%, respectively (all global and pairwise *p* < 0.001).

## 4. Discussion

In this multicenter cohort study of surgically treated patients with hip fractures, we identified three distinct early recovery trajectories of motor function using a growth mixture model. Approximately one-quarter of the patients belonged to the high-recovery class, one-third to the intermediate class, and more than 40% to the poor-recovery class. These trajectories were associated with baseline characteristics and discharge outcomes, highlighting the heterogeneous nature of recovery after hip fracture.

These findings are broadly in line with previous trajectory studies. Tseng et al. identified three distinct functional recovery patterns in older patients with hip fractures over a 2-year follow-up period, highlighting the influence of age and cognitive impairment [12]. Salpakoski et al. reported two heterogeneous mobility trajectories during a 10-week follow-up period and also presented entropy as a measure of classification quality [13]. More recently, Dakhil et al. analyzed longitudinal functional outcomes over 12 months in a hip fracture cohort from three Norwegian hospitals using a growth mixture model, identifying four distinct ADL trajectories [14]. Although the number of trajectories and follow-up duration differed, these studies collectively underscore the heterogeneous nature of post-fracture recovery. Compared with these studies, the strengths of the present work include the large multicenter sample across 17 hospitals, the focus on the acute postoperative phase (≤ 30 days), and the use of a comprehensive ADL measure (motor FIM) rather than walking status alone. By capturing early nonlinear changes, our study provides novel evidence of acute recovery heterogeneity in real-world clinical practice.

Patients in the poor-recovery class were more likely to be older, cognitively impaired, and to have trochanteric fractures and comorbidities, consistent with prior studies reporting that advanced age [4,6,8,25], cognitive impairment [4,5,6,26,27,28], and medical complications [4,6,29] predict poor rehabilitation outcomes. Conversely, the high recovery class was characterized by younger age, preserved cognition, and better pre-injury mobility, which facilitated rapid functional improvement. These findings underscore the importance of a comprehensive preoperative assessment in identifying patients at risk of delayed recovery.

Notably, early recovery patterns within the first 30 days were associated with functional independence and discharge destination. Patients in the poor-recovery class were more frequently discharged to rehabilitation or long-term care hospitals, whereas those in the high-recovery class were more often discharged home. These results suggest that early recovery trajectories may help inform clinical understanding of likely discharge pathways and the need for tailored rehabilitation, although this study did not aim to develop or evaluate predictive models.

Importantly, the results are robust to adjustments for hospital-level clustering. Introducing hospitals as a random effect did not materially alter the model fit, class proportions, or trajectory shapes. This finding suggests that recovery heterogeneity is primarily explained by patient-level characteristics rather than by institutional differences, reinforcing the generalizability of our findings across diverse acute care settings.

Nevertheless, significant differences in class distributions across hospitals were observed. While this may partly reflect institutional factors such as rehabilitation protocols or staffing, the proportions in smaller hospitals were more unstable, likely due to limited case numbers. These findings highlight the need for caution when interpreting hospital-level comparisons.

Moreover, the structure and duration of acute care and rehabilitation pathways may influence recovery trajectories. In our Japanese cohort, the median length of stay was relatively long (29 days), which likely allowed for greater in-hospital recovery and may have affected the shape and distribution of trajectory classes. In contrast, healthcare systems with shorter acute stays and earlier transitions to rehabilitation facilities (e.g., in the United States or parts of Europe) may exhibit different recovery patterns. These structural differences should be taken into account when applying our findings to other healthcare settings.

### Limitations

This study has several limitations. First, although data were collected from 17 hospitals, several potentially important factors—such as nutritional status, standardized frailty indices, and socioeconomic conditions—were not captured in the registry and therefore could not be incorporated into the analyses. These unmeasured confounders may have influenced both baseline functional status and subsequent recovery trajectories. Second, long-term functional recovery beyond 30 days was not examined, limiting the ability to evaluate whether early trajectories persist or diverge over time. Third, a substantial portion of patients (59.2%) lacked 28-day FIM-motor data, primarily due to early discharge or transfer. Because early discharge may occur for divergent reasons—either rapid improvement or limited potential for further recovery—this missingness may introduce bidirectional selection bias. To address this, we conducted a sensitivity analysis restricted to patients with complete 28-day data, which yielded highly consistent class structures and improved classification quality. Finally, as this was an observational study, causal inference remains limited despite adjustment for multiple covariates and hospital-level clustering.

In conclusion, we identified three distinct motor function recovery trajectories after hip fracture surgery. Although not designed as a prognostic model, these patterns were meaningfully associated with baseline characteristics and key outcomes. Future research should assess whether incorporating trajectory data can enhance predictive models for discharge planning and rehabilitation needs.

## Figures and Tables

**Table 1:** Baseline Characteristics, Functional Status, and Clinical Course of Hip Fracture Patients.

Variable	All (n = 2423)	Missing
Age (years)	87.00 [81.00, 92.00]	7
BMI (kg/m^2^)	20.00 [17.80, 22.40]	22
Sex-Male (%)	543 (22.4)	1
Cognitive impairment (%)	1201 (49.6)	16
Fracture type (%)		3
Neck	1230 (50.8)	
Intertrochanter	1190 (49.1)	
Pre-fracture living situation (%)		0
Own home	1818 (75.0)	
Nursing home	551 (22.7)	
Other hospital	54 (2.2)	
Pre-fracture indoor walking status (%)		1
Without aids	1234 (50.9)	
One-point cane	407 (16.8)	
Walker	468 (19.3)	
Other aids	93 (3.8)	
Wheelchair	220 (9.1)	
Pre-fracture outdoor walking status (%)		212
Without aids	882 (36.4)	
One-point cane	425 (17.5)	
Walker	282 (11.6)	
Other aids	49 (2.0)	
Wheelchair	573 (23.6)	
Mediation comorbidity		
Respiratory disease (%)	236 (9.7)	1
Cardiovascular disease (%)	606 (25.0)	1
Neurological disease (%)	391 (16.1)	1
Hospital stay (days)	29.00 [20.00, 46.00]	0
Waiting days for surgery (days)	2.00 [1.00, 4.00]	9
Waiting days for rehabilitation (days)	2.00 [1.00, 4.00]	14
Time to first walk in rehabilitation after surgery (days)	4.00 [3.00, 7.00]	299
Average rehabilitation time per day (minutes/day)	36.00 [26.00, 50.00]	39
FIM-Total (POD1)	44.50 [33.00, 54.00]	43
FIM-Motor (POD1)	18.00 [16.00, 23.00]	43
FIM-Total (POW1)	58.00 [41.00, 79.00]	109
FIM-Motor (POW1)	33.00 [22.00, 49.00]	109
FIM-Total (POW4)	76.00 [52.00, 96.00]	1434
FIM-Motor (POW4)	51.00 [32.00, 66.00]	1434
FIM-Total (POW8)	82.00 [58.00, 102.00]	2128
FIM-Motor (POW8)	56.00 [38.00, 72.50]	2128
FIM-Total (POW12)	87.00 [66.50, 104.50]	2352
FIM-Motor (POW12)	59.00 [44.50, 75.00]	2352
FIM-Total at discharge	79.00 [54.00, 100.00]	64
FIM-Motor at discharge	53.00 [33.00, 69.00]	65
Complications during hospitalization		
Fall (%)	105 (4.3)	222
Peripheral nerve palsy (%)	31 (1.3)	5
DVT (%)	129 (5.3)	3
Infection (%)	68 (2.8)	1
Walking status at discharge (%)		31
Without aids	119 (4.9)	
One-point cane	236 (9.7)	
Walker	832 (34.3)	
Other aids	26 (1.1)	
Wheelchair	1179 (48.7)	
Destination after discharge (%)		4
Own home	467 (19.3)	
Nursing home	479 (19.8)	
Rehabilitation hospital	1362 (56.2)	
Other hospital	79 (3.3)	
Others	32 (1.3)	

Patients’ demographic data. Data are presented as numbers (percentages) or medians (25th and 75th percentiles). BMI, body mass index; DVT, deep vein thrombosis; POD, post-operative day; POW, post-operative week.

**Table 2:** Baseline Characteristics and Pairwise Comparisons among Three Patient Classes with Hip Fracture.

Variable	Class1 (N = 782)	Class2 (N = 977)	Class3 (N = 640)	Class1vs2	Class1vs3	Class2vs3
Age (years)	82.00 [74.00, 88.00]	88.00 [83.00, 92.00]	90.00 [86.00, 94.00]	<0.001	<0.001	<0.001
BMI (kg/m^2^)	20.40 [18.22, 22.87]	19.80 [17.60, 22.20]	19.70 [17.50, 22.10]	<0.001	<0.001	0.513
Hospital stay (days)	25.00 [18.00, 40.00]	30.00 [20.00, 47.00]	31.00 [22.00, 49.00]	<0.001	<0.001	0.042
Sex-Male (%)	183 (23.4)	211 (21.6)	145 (22.7)	1	1	1
Cognitive impairment (%)	147 (18.8)	525 (53.7)	523 (81.7)	<0.001	<0.001	<0.001
Fracture type (%)						
Neck	493 (63.0)	481 (49.2)	248 (38.8)	<0.001	<0.001	<0.001
Intertrochanter	289 (37.0)	496 (50.8)	392 (61.3)			
Mediation comorbidity						
Respiratory disease (%)	72 (9.2)	103 (10.5)	59 (9.2)	1	1	1
Cardiovascular disease (%)	155 (19.8)	272 (27.8)	173 (27.0)	<0.001	<0.001	0.765
Neurological disease (%)	88 (11.3)	169 (17.3)	127 (19.8)	<0.001	<0.001	0.219
Pre-fracture living situation (%)						
Own home	725 (92.7)	723 (74.0)	353 (55.2)	<0.001	<0.001	<0.001
Nursing home	47 (6.0)	228 (23.3)	269 (42.0)			
Other hospital	10 (1.3)	26 (2.7)	18 (2.8)			
Pre-fracture indoor walking status (%)						
Without aids	614 (78.5)	436 (44.6)	172 (26.9)	<0.001	<0.001	<0.001
One-point cane	90 (11.5)	215 (22.0)	101 (15.8)			
Walker	58 (7.4)	228 (23.3)	177 (27.7)			
Other aids	11 (1.4)	39 (4.0)	42 (6.6)			
Wheelchair	9 (1.2)	59 (6.0)	148 (23.1)			
Pre-fracture outdoor walking status (%)						
Without aids	518 (66.2)	315 (32.2)	125 (19.5)	<0.001	<0.001	<0.001
One-point cane	137 (17.5)	224 (22.9)	83 (13.0)			
Walker	63 (8.1)	153 (15.7)	88 (13.8)			
Other aids	12 (1.5)	31 (3.2)	18 (2.8)			
Wheelchair	52 (6.6)	254 (26.0)	326 (50.9)			

Values are presented as median [interquartile range] for continuous variables and number (percentage) for categorical variables. *p*-values are shown for pairwise comparisons using appropriate statistical tests (Mann–Whitney *U* test for continuous variables, chi-square test for categorical variables). Significance was set at *p* < 0.05.

**Table 3:** Multinomial Logistic Regression Analyses of Factors Associated with Functional Recovery Speed after Hip Fracture Based on FIM-Motor Scores (Reference: Class 2—Intermediate Recovery).

Variable	OR_class1vs2	*p*_Value_class1vs2	OR_class3vs2	*p*_Value_class3vs2
(Intercept)	106.04 (28.29–397.43)		0.05 (0.01–0.25)	
Age (per year increase)	0.95 (0.94–0.96)	0.002	1.01 (1.00–1.03)	0.187
BMI (per kg/m^2^ increase)	1.35 (1.04–1.75)	0.024	0.77 (0.59–1.01)	0.057
Sex-Male	1.00 (0.97–1.03)	0.898	1.02 (0.99–1.06)	0.256
Cognitive impairment—Yes	0.38 (0.29–0.49)	<0.001	2.80 (2.14–3.66)	<0.001
Fracture type [reference: neck]				
Intertrochanter	0.81 (0.65–1.01)	0.061	1.36 (1.09–1.70)	0.007
Respiratory disease—Yes	0.97 (0.68–1.40)	0.869	0.82 (0.57–1.18)	0.285
Cardiovascular disease—Yes	0.84 (0.65–1.08)	0.178	0.96 (0.75–1.23)	0.746
Neurological disease—Yes	0.62 (0.45–0.86)	0.004	1.16 (0.87–1.55)	0.314
Residence [reference: Own home]				
Nursing home	0.55 (0.37–0.81)	0.003	1.13 (0.86–1.48)	0.378
Other hospital	0.53 (0.23–1.19)	0.130	0.92 (0.47–1.79)	0.807
Pre-fracture indoor walking status [reference: without aids]				
One-point cane	0.52 (0.35–0.77)	0.001	1.30 (0.84–2.01)	0.239
Walker	0.51 (0.31–0.82)	0.007	1.43 (0.93–2.21)	0.105
Other aids	0.47 (0.21–1.07)	0.069	2.54 (1.38–4.68)	0.003
Wheelchair	0.42 (0.18–1.00)	0.047	3.81 (2.30–6.31)	<0.001
Pre-fracture outdoor walking status [reference: without aids]				
One-point cane	0.76 (0.53–1.09)	0.136	0.68 (0.43–1.10)	0.108
Walker	0.76 (0.47–1.22)	0.259	0.84 (0.51–1.40)	0.499
Other aids	0.81 (0.36–1.82)	0.610	0.52 (0.24–1.13)	0.098
Wheelchair	0.68 (0.41–1.14)	0.139	0.97 (0.61–1.55)	0.898

Odds ratios (ORs) with 95% confidence intervals (CI) and *p*-values are shown for comparisons between Class 1 vs. Class 2 and Class 3 vs. Class 2, based on a multinomial logistic regression model. **Class 1 (Early Recovery):** Patients with rapid functional improvement based on FIM-Motor trajectory; **Class 2 (Intermediate Recovery):** Reference group with moderate recovery speed; **Class 3 (Slow Recovery):** Patients with delayed or limited functional recovery; Categorical variables were coded with the following reference categories:; **Cognitive impairment:** No impairment; **Fracture type:** Femoral neck fracture; **Residence before fracture:** Own home; **Pre-fracture indoor/outdoor walking status:** Without aids; **Sex:** Female; **Comorbidities:** Absence of each respective condition. Continuous variables (e.g., age, BMI) are modeled per unit increase. A *p*-value < 0.05 is considered statistically significant.

**Table 4:** Functional Outcomes, Discharge Destinations, and Mobility Status by Functional Recovery Class Based on FIM-Motor Scores.

Variable	Class1 (N = 782)	Class2 (N = 977)	Class3 (N = 640)	Class1vs2	Class1vs3	Class2vs3
FIM-Motor at discharge	68.00 [58.00, 79.00]	48.50 [38.00, 62.00]	25.00 [19.00, 36.00]	<0.001	<0.001	<0.001
FIM-Total at discharge	100.00 [87.00, 113.00]	74.00 [60.25, 92.00]	43.00 [31.00, 60.00]	<0.001	<0.001	<0.001
Destination after discharge (%)						
Own home	257 (32.9)	151 (15.5)	50 (7.8)	<0.001	<0.001	<0.001
Nursing home	46 (5.9)	196 (20.1)	227 (35.5)			
Rehabilitation hospital	468 (59.8)	592 (60.8)	300 (46.9)			
Other hospital	5 (0.6)	21 (2.1)	52 (8.1)			
Others	6 (0.8)	14 (1.4)	11 (1.7)			
Walking status at discharge (%)						
Without aids	89 (11.5)	19 (2.0)	10 (1.6)	<0.001	<0.001	<0.001
One-point cane	180 (23.2)	53 (5.5)	0 (0.0)			
Walker	367 (47.2)	372 (38.4)	85 (13.6)			
Other aids	8 (1.0)	16 (1.7)	2 (0.3)			
Wheelchair	133 (17.1)	509 (52.5)	527 (84.5)			

Values are presented as median [interquartile range] for continuous variables and number (percentage) for categorical variables. *p*-values correspond to pairwise comparisons between recovery classes (Class 1 vs. 2, Class 1 vs. 3, and Class 2 vs. 3), using the Mann–Whitney *U* test for continuous data and chi-square test for categorical data. **Class 1 (Early Recovery):** Patients with high FIM-Motor scores at discharge, indicating rapid functional improvement; **Class 2 (Intermediate Recovery):** Patients with moderate FIM-Motor scores; **Class 3 (Slow Recovery):** Patients with low FIM-Motor scores, indicating delayed recovery. **Abbreviations: FIM-Motor:** Functional Independence Measure—Motor subscale; **FIM-Total:** Functional Independence Measure—Total score; Discharge destinations and walking status reflect patients’ condition at the time of discharge. A *p*-value < 0.05 is considered statistically significant.

## Data Availability

The data are available from the corresponding author upon reasonable request due to privacy and ethical restrictions.

## Supplementary Materials

The following supporting information can be downloaded at: https://www.mdpi.com/article/10.3390/geriatrics10060167/s1, Figure S1. Distribution of FIM motor trajectory classes across hospitals with corresponding case counts. Each bar represents one hospital, and the proportion of each trajectory class is shown. Green (Class 1): High-functioning trajectory group. Blue (Class 2): Moderate-functioning trajectory group. Red (Class 3): Low-functioning trajectory group. Table S1. Latent Class Model Fit Statistics and Class Distribution.

## Abbreviations

The following abbreviations are used in this manuscript:

ADL	Activities of Daily Living
APP	Average Posterior Probability
BIC	Bayesian Information Criterion
DDLIS-PD	Degree of Daily Life Independence Score for People with Dementia
FIM	Functional Independence Measure
GMM	Growth Mixture Modeling
LOS	Length of Stay
OR	Odds Ratio
STROBE	Strengthening the Reporting of Observational Studies in Epidemiology
