# Supplementary material for: Recovery Trajectories of Motor Function After Hip Fracture Surgery in Older Patients: A Multicenter Growth Mixture Modeling Study in Acute Care Hospitals
Source: Geriatrics (Basel). 2025 Dec 15;10(6):167. doi: 10.3390/geriatrics10060167 (PMC12732734; doi:10.3390/geriatrics10060167)
Supplement: Supplementary file 1 [file geriatrics-10-00167-s001.zip › Supplementary_material-edited.pdf]

Supplementary Figure 1. Distribution of FIM motor trajectory classes across hospitals with corresponding case counts.

Each bar represents one hospital, and the proportion of each trajectory class is shown.

Green (Class 1): High-functioning trajectory group.

Blue (Class 2): Moderate-functioning trajectory group.

Red (Class 3): Low-functioning trajectory group.
