# Supplementary material for: Recovery Trajectories of Motor Function After Hip Fracture Surgery in Older Patients: A Multicenter Growth Mixture Modeling Study in Acute Care Hospitals
Source: Geriatrics (Basel). 2025 Dec 15;10(6):167. doi: 10.3390/geriatrics10060167 (PMC12732734; doi:10.3390/geriatrics10060167)
Supplement: Supplementary file 1 [file geriatrics-10-00167-s001.zip › SupplTable1-edited.pdf]

Suppl Table 1. Latent Class Model Fit Statistics and Class Distribution.

A. Model Fit Indices

Model Name	No. of Classes	Log-likelihood	BIC	Entropy
1-class model	1	–28108.9	56272.3	—
2-class model	2	–27182.9	54459.2	—
3-class model	3	–26988.9	54110.1	0.607
3-class model (Facility-adjusted)	3	–26,870.2	53890.4	0.623
4-class model	4	–27090.5	54408.1	—
3-class model (Sensitivity analysis)	3	-21500.5	45118.3	0.676

The 3-class model showed the lowest BIC and acceptable entropy (>0.6), supporting its selection as the final model.

The "3-class model (Sensitivity analysis)" refers to a growth mixture model restricted to individuals with available motor FIM data at postoperative day 28 (n = 989). The model structure is otherwise identical to the primary analysis.

B. Class Distribution and Average Posterior Probabilities (APP)

Class	n	%	APP
Class 1 (Early Recovery)	782	32.6%	0.901
Class 2 (Intermediate Recovery)	977	40.7%	0.825
Class 3 (Slow Recovery)	640	26.7%	0.833

APP: Average posterior probability for each class assignment, indicating classification precision.

C. Class Distribution and Average Posterior Probabilities (APP) in Sensitivity Analysis

Class	n	%	APP
Class 1 (Early Recovery)	312	31.5%	0.844
Class 2 (Intermediate Recovery)	402	40.7%	0.835
Class 3 (Slow Recovery)	275	27.9%	0.883

The sensitivity analysis was restricted to individuals with available motor FIM data at postoperative day 28 (n = 989). The model specification was identical to the primary model, with latent class trajectory modeling limited to the first 30 days after surgery.
