# Supplementary figures and images for: Recovery Trajectories of Motor Function After Hip Fracture Surgery in Older Patients: A Multicenter Growth Mixture Modeling Study in Acute Care Hospitals
Source: Geriatrics (Basel). 2025 Dec 15;10(6):167. doi: 10.3390/geriatrics10060167 (PMC12732734; doi:10.3390/geriatrics10060167)

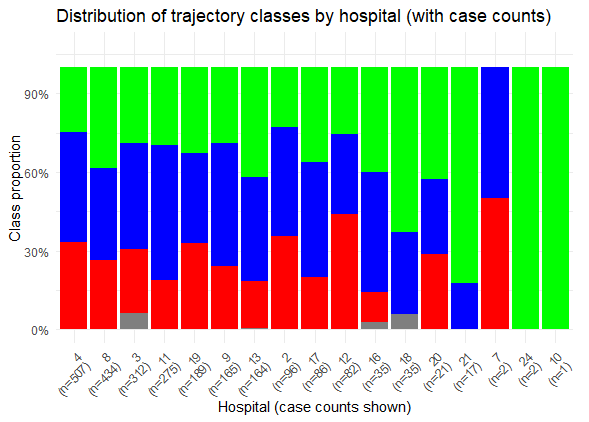

Supplement: Supplementary file 1 [file geriatrics-10-00167-s001.zip › SupplFigure1.png]
